# New insights into the evolutionary history of biological nitrogen fixation

**DOI:** 10.3389/fmicb.2013.00201

**Published:** 2013-08-05

**Authors:** Eric S. Boyd, John W. Peters

**Affiliations:** Department of Chemistry and Biochemistry and Department of Microbiology, Montana State UniversityBozeman, MT, USA

**Keywords:** nitrogen fixation, great oxidation event, NIf, methanogens

## Abstract

Nitrogenase, which catalyzes the ATP-dependent reduction of dinitrogen (N_2_) to ammonia (NH_3_), accounts for roughly half of the bioavailable nitrogen supporting extant life. The fundamental requirement for fixed forms of nitrogen for life on Earth, both at present and in the past, has led to broad and significant interest in the origin and evolution of biological N_2_ fixation. One key question is whether the limited availability of fixed nitrogen was a factor in life's origin or whether there were ample sources of fixed nitrogen produced by abiotic processes or delivered through the weathering of bolide impact materials to support this early life. If the latter, the key questions become what were the characteristics of the environment that precipitated the evolution of this oxygen sensitive process, when did this occur, and how was its subsequent evolutionary history impacted by the advent of oxygenic photosynthesis and the rise of oxygen in the Earth's biosphere. Since the availability of fixed sources of nitrogen capable of supporting early life is difficult to glean from the geologic record, there are limited means to get direct insights into these questions. Indirect insights, however, can be gained through phylogenetic studies of nitrogenase structural gene products and additional gene products involved in the biosynthesis of the complex metal-containing prosthetic groups associated with this enzyme complex. Insights gained from such studies, as reviewed herein, challenge traditional models for the evolution of biological nitrogen fixation and provide the basis for the development of new conceptual models that explain the stepwise evolution of this highly complex life sustaining process.

## Introduction

All life requires fixed sources of nitrogen (N) and its availability is what often limits productivity in natural systems (Falkowski, 1997). Most N on Earth is in the form of dinitrogen (N_2_), which is not bio-available. On early Earth, fixed sources of N may have been supplied by abiotic processes such as electrical (i.e., lightning) based oxidation of N_2_ to nitric oxide (NO) (Yung and McElroy, 1979; Kasting and Walker, 1981) or mineral (e.g., ferrous sulfide) based reduction of N_2_ (Schoonen and Xu, 2001; Summers et al., 2012), nitrous oxide (Summers et al., 2012), or nitrite (NO^−^_2_)/nitrate (NO^−^_3_) (Summers, 2005; Singireddy et al., 2012) to NH_3_. Abiotic sources of fixed N (e.g., NO, NO^−^_2_, NO^−^_3_, NH_3_) are thought to have become limiting to an expanding global biome (Kasting and Siefert, 2001; Navarro-González et al., 2001), which may have precipitated the innovation of biological mechanisms to reduce N_2_.

The primary enzyme that catalyzes the reduction of N_2_ to bio-available NH_3_ today is the molybdenum (Mo)-dependent nitrogenase (Nif) although other phylogenetically-related forms of nitrogenase that differ in their active site metal composition (termed alternative nitrogenase, or Vnf & Anf) may also contribute NH_3_ in environments that are limiting in Mo (Joerger and Bishop, 1988; Kessler et al., 1997). Nitrogenase catalyzes the production of half, if not more, of all of the fixed nitrogen on Earth today (Falkowski, 1997). As such, this process functions to relieve fixed N limitation in natural ecosystems (Zehr et al., 2003) and is likely to have a disproportionate effect on the functioning of an ecosystem, relative to inputs from other populations. Thus, organisms which fix nitrogen in natural communities have been described as keystone species (Hamilton et al., 2011a).

## Taxonomy, phylogeny, and physiology of organisms that fix dinitrogen (N_2_)

The taxonomic distribution of nitrogenase is curiously restricted to bacteria and archaea, with no known examples of the genes encoding for this process occurring within the eukarya (Raymond et al., 2004; Boyd et al., 2011a; Dos Santos et al., 2012). Within the archaea, nitrogenase has a narrow distribution and is restricted to methanogens (Euryarcheota) within the orders *Methanococcales*, *Methanobacteriales*, *Methanosarcinales* and has yet to be identified among members of the Crenarchaeota, Thaumarchaeota, or Nanoarchaeota. Likewise, *nif* exhibits a limited distribution among bacteria. For example, *nif* has been identified in a number of aerobic soil bacteria and has been identified in the genomes of 21 of the 44 sequenced cyanobacterial genomes, including those that inhabit terrestrial (e.g., *Cyanothece* and *Synechococcus* strains) and marine (*Crocosphaera watsonii*) environments. In addition, *nif* gene clusters are commonly detected in the genomes of *Firmicutes*, *Chloroflexi*, *Chlorobi*, and *Bacteroidetes* and in several lineages of *Actinobacteria* and *Proteobacteria*.

N_2_ fixation is associated with a diversity of microorganisms that display a wide variety of physiologies that range from obligate aerobes to obligate anaerobes (Raymond et al., 2004; Boyd et al., 2011a; Dos Santos et al., 2012). Since nitrogenase is very sensitive to oxygen (Gallon, 1981), different classes of aerobic or facultative anaerobic organisms have evolved a number of mechanisms to perform N_2_ fixation in an otherwise oxic environment. These mechanisms will only be treated briefly in this article and the reader is referred to extensive reviews written previously that focus on this topic (Gallon, 1981; Berman-Frank et al., 2003). Probably the most recognized mechanism for fixing N_2_ in an oxic environment is associated with symbiotic nitrogen fixation in which plants provide a microaerobic niche where oxygen tensions are maintained at low levels by a high affinity oxygen binding protein known as leghemoglobin, which is produced by the host plant (Ott et al., 2005). This strategy of O_2_ sequestration allows the symbiotic diazotroph (e.g., Rhizobia) to maintain aerobic respiration while catalyzing O_2_ sensitive N_2_ fixation. In addition, nitrogen fixation occurs under anoxic conditions in strict anaerobes and only during periods of anaerobic growth in facultative anaerobes. Cyanobacteria, the only diazotrophic lineage that produces molecular O_2_ as a product of its metabolism, have developed a number of mechanisms to fix N_2_ (Fay, 1992; Berman-Frank et al., 2003). For example, non-filamentous cyanobacteria tend to operate on a diurnal cycle where N_2_ fixation is up-regulated at night when oxygen tensions have dropped due to concomitant decreases in the production of photosynthetic O_2_ and increased O_2_ consumption by co-inhabiting heterotrophic populations. Alternatively, the co-occurrence of N_2_ fixation and O_2_ production in filamentous cyanobacteria is made possible by spatial segregation of nitrogenase in anaerobic heterocyst structures where increased protection of the nitrogenase complex is achieved through the photoreduction of O_2_ to H_2_O in photosystem I (Milligan et al., 2007), also known as the Mehler reaction (Mehler, 1957). In contrast, in obligate aerobes the nitrogen fixation apparatus is protected by what has been described as a cytochrome-dependent respiratory protection mechanism whereby high rates of respiration ensure the consumption of oxygen at the cell membrane thereby maintaining low intracellular oxygen tensions (Poole and Hill, 1997). It is likely that these mechanisms emerged later in the evolutionary history of biological nitrogen fixation due to the increased complexity of *nif* gene clusters associated with microorganisms adapted to fixing nitrogen in an oxygenated atmosphere (Boyd et al., 2011a,b; Dos Santos et al., 2012). The simplest assemblages of specific genes associated with nitrogen fixation occur in strict anaerobes. Nevertheless, tracing the evolutionary trajectory of this process and identifying the most ancient nitrogen fixers present in extant biology has been a challenge.

### How ancient is biological nitrogen fixation?

Biological nitrogen fixation has been suggested to be an ancient and perhaps even primordial process (Falkowski, 1997; Fani et al., 2000). This prevailing view is based on simulations of Archaean atmospheric chemistry that contend that decreasing CO_2_ concentrations and concomitant decreases in abiotic N_2_ oxidation to NO led to a nitrogen crises at ~3.5 Ga (Kasting and Siefert, 2001). However, using the same logic, Navarro-González argue that the nitrogen crisis could have ensued much later, even as late as 2.2 Ga (Navarro-González et al., 2001). Abiotic sources of nitrogen produced through mechanisms such as lightning discharge or mineral based catalysis (Yung and McElroy, 1979; Schoonen and Xu, 2001) are thought to have become limiting to an expanding global biome. Since extant nitrogenase functions to relieve N limitation in ecosystems (Zehr et al., 2003; Rubio and Ludden, 2008), the imbalance in the supply and demand for fixed N is thought to have represented a strong selective pressure that may have precipitated the emergence of nitrogen fixation (Raymond et al., 2004; Boyd et al., 2011a). Little direct evidence exists, however, with respect to the availability of ammonia or other reduced forms of nitrogen over the course of geological time, although several recent isotopic analyses of shale kerogens have suggested ample enough supply of ammonia to support nitrifying populations in the late archean, >2.5 Ga (Garvin et al., 2009; Godfrey and Falkowski, 2009).

**Figure 1:**
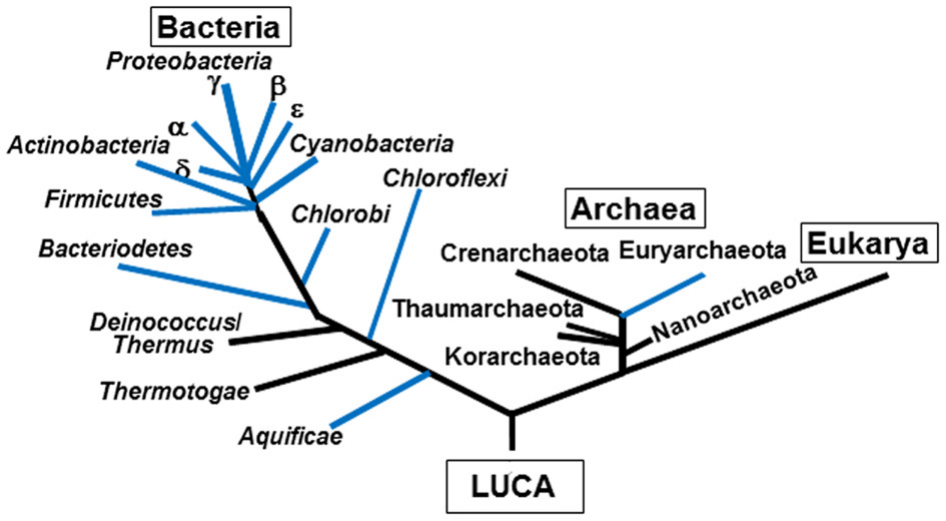
**A schematic of a 3 domain taxonomic tree of life with lineages that include nitrogen fixing organisms, as identified through genome screening for *nifHDKENB*, overlaid in blue**.

While the geologic record cannot yet definitively reconcile when fixed sources of nitrogen became limiting, one can ask the general question of whether the overall distribution and phylogenetic history of nitrogenase and its associated functionalities in extant biology are consistent with a primordial process or a property of the **Last Universal Common Ancestor (LUCA)**. Although widely distributed among bacteria, the distribution of the process is far from universal among archaea, and as previously mentioned has never been identified among members of the eukarya (Boyd et al., 2011a; Dos Santos et al., 2012). Moreover, unlike processes and functionalities that we ascribe to properties of LUCA, nitrogenase is not generally (note caveat below) associated with deeply rooted lineages identified by 16S ribosomal RNA evolutionary trajectories (Figure 1).

**Figure 2:**
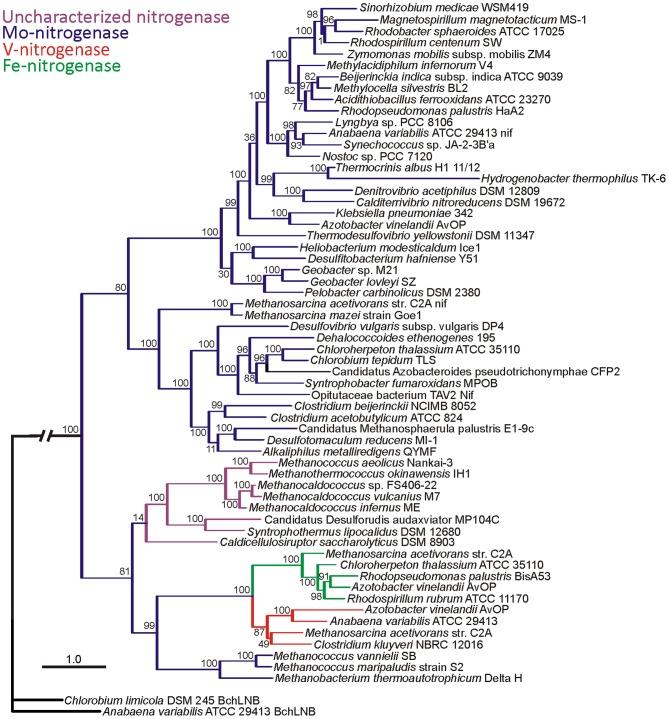
**Maximum-likelihood phylogenetic reconstruction of a concatenation of Nif/Anf/Vnf and uncharacterized HDK protein sequences.** Individual protein sequences were aligned, concatenated, and subjected to evolutionary reconstruction as described previously (Boyd et al., 2011b). The metal composition of the nitrogenase active site clusters are overlaid in blue (Nif), purple (“uncharacterized nitrogenase”), red (Vnf), and green (Anf). Bootstrap values are indicated at the nodes. Concatenations of paralogous proteins involved in the synthesis of chlorophyll/bacteriochlorophyll (Bch/ChlLNB) were used to root the phylogeny. The hash at the root was introduced to conserve space.

Our recent screening of two representative *Aquificales* genomes [i.e., *Thermocrinis albus* (Wirth et al., 2010) and *Hydrogenobacter thermophilus* (Zeytun et al., 2011)] reveal the presence of nitrogenase gene clusters. The identification of *nif* gene clusters in the genomes of thermophilic members of the *Aquificales*, regarded by many as the most deeply rooted bacterial lineage (Reysenbach et al., 2005), prompted a re-analysis of the distribution of *nif* on a depiction of the taxonomic tree of life (Figure 1). Although this analysis suggests that deeply rooted bacteria encode for *nif* (e.g., *Aquificales*) the limited distribution of *nif* among deeply branching archaea (e.g., Thaumarchaeota, Nanoarchaeota lineages) and deeply branching bacteria (Thermus/Deinococcus) suggests that *nif* may have been subject to extensive gene loss/lateral gene transfer or was not a property of the Last Universal Common Ancestor (LUCA). If Nif was a property of LUCA, then phylogenetic analyses of *nif* gene or protein sequences, would be expected to reveal reciprocally **monophyletic** bacterial and archaeal lineages (e.g., subtrees containing just archaeal homologs and bacterial homologs joined at LUCA). However, our previous maximum likelihood and Bayesian phylogenetic analyses of a concatenation of the structure proteins required for nitrogen fixation (homologs of H, D, and K, described below) indicate that archaea are **paraphyletic** with respect to bacteria (Boyd et al., 2011a,b), suggesting that Nif emerged after the divergence of archaea and bacteria. Additionally, our current maximum likelihood analysis of a concatenated HDK protein alignment block (Figure 2) indicates that Nif proteins from deeply rooted thermophilic members of the *Aquificales* were acquired recently through a **lateral gene transfer** with a more recently evolved and thermophilic member of the bacterial phylum *Deferribacteres* (e.g., ancestor of *Calditerrivibrio nitroreducens* or *Denitrovibrio acetiphilus*) (Figure 2). This suggests that *Aquificales* acquired *nif* in the recent evolutionary past from an exchange with a bacterial partner in a thermal environment. In further support of this hypothesis, numerous *Aquificales* genera (e.g., *Hydrogenobaculum*) do not encode *nif* (Romano et al., 2013), despite branching more basal than *Thermocrinis* and *Hydrogenobacter* in 16S rRNA gene phylogenetic reconstructions (Eder and Huber, 2002). *Hydrogenobaculum* spp. tend to populate acidic geothermal environments where NH^+^_4_ produced from magmatic degassing is in much higher supply (Holloway et al., 2011) whereas *Thermocrinis* and *Hydrogenobacter* tend to populate circumneutral to alkaline environments that are N limited (Reysenbach et al., 2005). Thus, the recent diversification of *Aquificales* into N limited environments may have been facilitated by acquisition of *nif*. Together, these findings add to a growing body of evidence suggesting that lateral gene transfer has played a significant role in expanding the taxonomic and ecological distribution of N_2_ fixation (Raymond et al., 2004; Kechris et al., 2006; Bolhuis et al., 2009).

In spite of the uncertainty and controversy that surrounds molecular dating techniques based on phylogenetic reconstructions (Bromham and Penny, 2003; Rutschmann, 2006), our recent data-driven attempt at addressing the age of nitrogen fixation using molecular dating techniques places its origin within a window of ~1.5–2.2 Ga (Boyd et al., 2011a). This time frame corresponds to a period of earth history where inferred fixed N levels are thought to have become limiting, O_2_ concentrations began to increase, and dissolved molybdenum (Mo) concentrations started to increase (Navarro-González et al., 2001; Anbar and Knoll, 2002; Berman-Frank et al., 2003; Canfield, 2005; Anbar et al., 2007; Anbar, 2008). Many of the geochemical changes associated with this period of geological history are likely a consequence of the production of oxygen by proliferating populations of oxygenic phototrophs (Anbar and Knoll, 2002; Anbar, 2008). The production of oxygen may have opened up new ecological niches, allowing the global biome to diversify and radiate into new environmental realms. This expansion of the biosphere would have created additional demand on the bioavailable N pool and may have increased the selective pressure to evolve a biological mechanism to increase the local bioavailable N pool.

### What are the most deeply rooted extant organisms that harbor nitrogenase?

To answer this question one must first define a set of criteria for the minimum number of genes that are required to catalyze N_2_ fixation in extant organisms. This is not as simple as it is in many other enzyme systems, since just the presence or absence of genes that encode the structural protein are insufficient to produce an active Mo-dependent nitrogenase. Rather, a series of additional genes are required to synthesize the complex iron-molybdenum cofactor (FeMo-co) located at the active site of nitrogenase (Rubio and Ludden, 2008). From previous genomic, biochemical, and molecular genetic studies of different microbial sources, we and others have established a set of criteria for biological nitrogen fixation that requires at a minimum the structural genes *nifH, nifD*, and *nifK* and three additional FeMo-cofactor biosynthetic genes *nifE, nifN*, and *nifB* (Boyd et al., 2011a; Dos Santos et al., 2012). This criteria is based primarily on deletion mutation analysis of *nifEN* (Ugalde et al., 1984; Jacobson et al., 1989; Roll et al., 1995; Hu et al., 2005) and *nifB* (Shah et al., 1994; Christiansen et al., 1998) which result in the production of an inactive and FeMo-cofactor-less nitrogenase. Moreover, *nif* clusters of all sequenced nitrogen fixers that have been characterized (note caveat with respect to “uncharacterized nitrogenase,” as discussed below) have at a minimum these six gene products (*nifHDKENB*) (Boyd et al., 2011a; Dos Santos et al., 2012). Using these criteria, we exploited specific genetic events involved in the evolution of these six proteins, with particular attention paid to those that are involved in the biosynthesis of the active site cluster, in order to identify which extant organism harbors the oldest nitrogenase.

**Figure 3:**
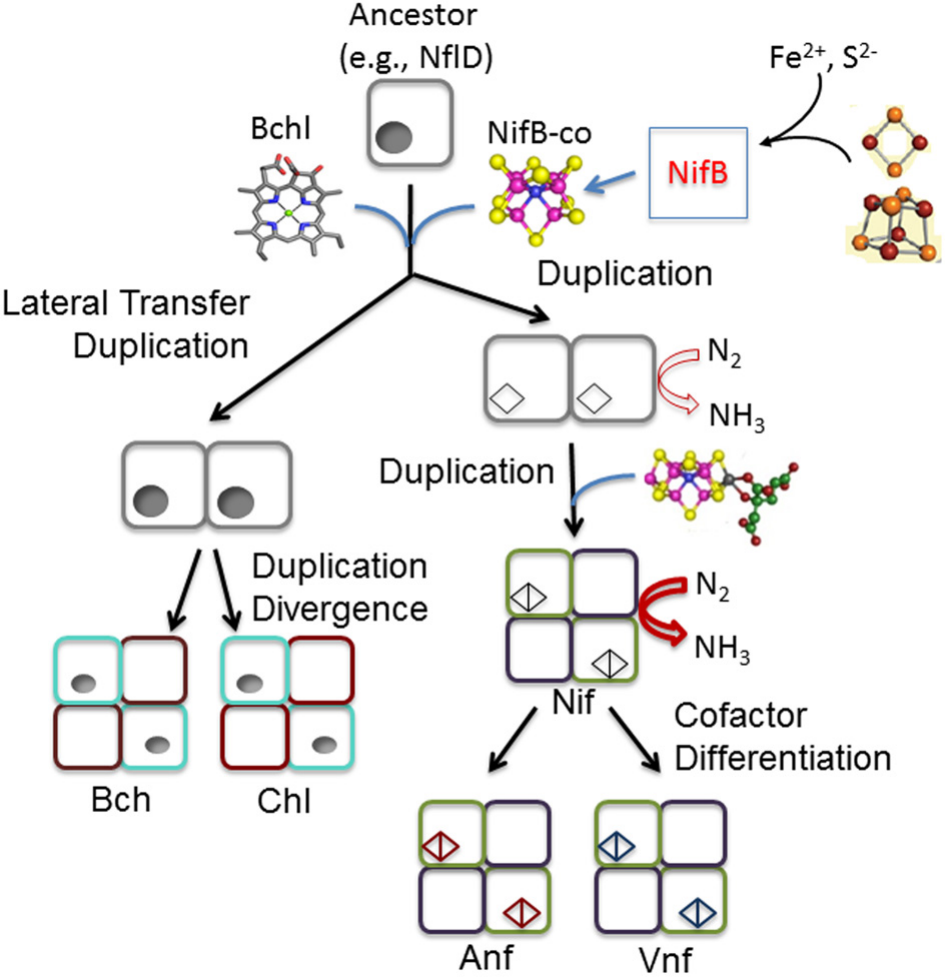
**Hypothetical scheme depicting the evolution of nitrogenase from its protein ancestor. Parsimony** suggests that the likely ancestor of these protein complexes was a NflD-like protein present in an ancestral methanogen. The movement of an ancestor of a NflD-like protein to anoxygenic phototrophs, and the diversification of this protein into BchN, would necessitate lateral gene transfer followed by a duplication event. In contrast, vertical inheritance of a duplicated NflD ancestor in a methanogen can account for proto NifD. The diversification of the duplicated NflD like ancestor into a proto homodimeric NifD (i.e., protonitrogenase) is presumed to have been precipitated by interaction with an ancestor of the radical SAM protein NifB, which in extant biology catalyzes the formation of the FeMo-co precursor, NifB-co, from simple FeS clusters. Here, NifB-co or the like could have serendipitously been inserted in the open active site cavity presumed to be present in the protonitrogenase ancestor (e.g., BchN- or NflD-like) conferring the ability to perhaps catalyze a low level of N_2_ reduction. A second duplication of *nifD* and the subsequent diverisification of this gene (loss of FeMo-co binding site) led to *nifK*. The later bicistronic duplication of *nifDK* and subsequent diversification of these genes to *nifEN* yielded the ability to further mature biosynthetic intermediates into FeMo-co. In this depiction, metal cofactor binding sites within proteins are indicated by lobes whereas those that likely bind organic cofactors (e.g., protochlorophyllide) are indicated by circles. Open lobes depict sites where a cluster similar to NifB-co may have been bound by a protonitrogenase.

The first relationship that was exploited was that between the structural genes, *nifDK* that encode the MoFe protein (NifDK) and the paralogous genes, *nifEN* that encode a scaffold complex (NifEN) that functions in FeMo-cofactor biosynthesis. Primary amino acid sequence comparisons of NifD, NifK, NifE, and NifN reveal significant homology indicating that these gene products evolved from a common ancestor (Brigle et al., 1987; Fani et al., 2000; Raymond et al., 2004; Soboh et al., 2010; Boyd et al., 2011a). It has been suggested that three independent **gene duplications** yielded these four related gene products (Fani et al., 2000). As depicted in Figure 3, the first duplication of an ancestor of *nflD*-like common ancestor resulting in a protein that over time diversified to form *nifD*. *nifD* was then duplicated and through subsequent diversification formed *nifK*, resulting in the heterotetrameric MoFe protein, NifDK (Fani et al., 2000; Raymond et al., 2004; Boyd et al., 2011a). Subsequently, a bicistronic duplication of *nifDK* is thought to have yielded *nifEN*. Phylogenetic reconstructions of NifD, NifK, NifE, and NifN reveal that NifE and NifN sequences are nested among NifD and NifK sequences, respectively, consistent with this evolutionary trajectory (Boyd et al., 2011a). The most basal branching NifD, NifK, NifE, and NifN sequences are associated with hydrogenotrophic methanogens (*Methanobacteriales*, *Methanococcales*), suggesting this physiological class of organism to be most representative of the ancestor of Nif. This finding was further supported by a phylogenetic analysis of a concatenation of NifHDK, which revealed this class of organism as the basal branch (Figure 2) (Boyd et al., 2011b). Importantly, the other early branching lineages of nitrogenase all derive from obligate anaerobes (Figure 2) (Boyd et al., 2011a,b), suggesting that this process emerged in an anoxic environment, consistent with the oxygen sensitivity of this enzyme (Rubio and Ludden, 2008). Moreover, these findings are consistent with our suggestion that the mechanisms organisms evolved to fix nitrogen in the presence of oxygen are more recent evolutionary innovations.

The evolutionary history of NifB reinforces results implicating hydrogenotrophic methanogens as the oldest nitrogen-fixing organisms. NifB is a radical generating S-adenosylmethionine dependent enzyme that is involved in generating the hexacoordinated carbide at the center of the FeMo-cofactor (Wiig et al., 2012), the presumed key structural determinant of nitrogenase function. All Mo-nitrogenase systems identified to date encode for NifB, which is consistent with its essential role in the synthesis of the active site cofactor and with its presumed central role in the origin of nitrogenase (Figure 3) (Soboh et al., 2010; Boyd et al., 2011a,b). Most NifB proteins associated with extant organisms exist as two domain proteins composed of a radical SAM functionality (SAM domain only) and a putative carrier protein functionality (NifX domain) resulting from a **fusion** of a gene encoding the core SAM domain and a standalone gene, *nifX* (Rubio and Ludden, 2008). Phylogenetic reconstruction of just the SAM domain indicate that the fusion of the gene encoding this domain with *nifX* is a recent evolutionary innovation (Boyd et al., 2011a). Methanogens, which harbor *nifB* homologs that diverged prior to the fusion with the *nifX* domain, branch at the base of the NifB-SAM domain tree (Boyd et al., 2011a). The fact that methanogens and other early descendants on the NifB phylogeny (e.g., firmicutes, chloroflexi) are strict anaerobes provide additional support that Mo-nitrogenase had its origin in an anoxic environment.

### Are alternative nitrogenases evolutionary ancestors of Mo-nitrogenase?

Although the majority of present-day biological N_2_ reduction is catalyzed by Nif (Rubio and Ludden, 2008), alternative forms exist with active site cofactors that lack Mo and contain vanadium and iron (V-nitrogenase) or iron only (Fe-only nitrogenase) (encoded by *vnf* and *anf*, respectively). The evolutionary trajectory of the different metal containing nitrogenases has been of keen interest since their discovery nearly 30 years ago. It has been suggested that nitrogen fixation by Vnf or Anf might have preceded Nif prior to the *Great Oxidation Event* and the advent of oxygenic photosynthesis ~2.5–2.8 Ga (Anbar and Knoll, 2002; Raymond et al., 2004). This proposal was based on chemostratigraphic measurements that indicate limited Mo under the reducing environment of the early Earth (Anbar et al., 2007) where most Mo would have existed as complexes of insoluble Mo-sulfides (Helz et al., 1996). Although this logic is sound and the potential for alternative nitrogenases as ancestors of Mo-nitrogenase is a rational hypothesis, there are a number of observations that suggest that this is not likely to be the case.

Alternative nitrogenases occur in a small number of organisms and to date have never been identified in taxa that do not also encode a Mo-nitrogenase (Boyd et al., 2011a). Moreover, gene clusters encoding the structural components of the alternative nitrogenases possess only a fraction of the cofactor biosynthetic genes required for FeMo-cofactor biosynthesis (Boyd et al., 2011a,b), implying a dependence on *nif*-encoded biosynthetic machinery. Indeed, targeted and global transcriptional analyses of the model nitrogen-fixing organism, *Azotobacter vinelandii*, indicates that the synthesis of an active alternative nitrogenase (Vnf- or Anf) requires the expression of a number of FeMo-cofactor synthetic gene products encoded by genes in *nif* clusters (Wolfinger and Bishop, 1991; Hamilton et al., 2011b). Finally, extant Mo-containing forms of nitrogenase are significantly more efficient at binding N_2_ and reducing it to ammonia than V- and Fe-only nitrogenase (Joerger and Bishop, 1988; Eady, 1996), and would have presumably been highly selected for under conditions of fixed N limitation that are thought to have characterized ecosystems at this time (Navarro-González et al., 2001; Berman-Frank et al., 2003). These observations make it difficult to rationalize an ancestry whereby Mo-nitrogenase arose from an alternative nitrogenase without invoking extensive gene loss and/or significant genomic rearrangement.

The gene clusters associated with alternative nitrogenase encode for only the structural proteins (HDK) and generally lack homologs of key biosynthetic genes (ENB). Exceptions where biosynthetic genes are also encoded in alternative gene clusters include the *vnf* operon in *A. vinelandii* and *Rhodopseudomonas palustris* CGA009 which encode for EN, although these copies are the result of a recent duplication of *nifEN* in these taxa (Boyd et al., 2011a). Thus, extant alternative nitrogenase operons do not encode for the complement of genes required to independently synthesize an active nitrogenase, and thus do not meet the criteria set forth above. Phylogenetic placement of alternative nitrogenase in the evolution of nitrogenase based on structural genes (D or K) has resulted in ambiguous results with respect to which form of nitrogenase is ancestral (Boyd et al., 2011a). Likewise, phylogenetic analyses of Anf/Vnf/NifD alone or concatenations of Anf/Vnf/NifHD also lead to ambiguous results (Raymond et al., 2004). Since all three nitrogenase (Mo-, V-, and Fe-only) have dedicated structural proteins (H, D, and K), we recently conducted a phylogenetic study of a concatenation of these three protein sequences (Boyd et al., 2011b). The well-resolved and strongly supported phylogenetic reconstruction (Figure 2) indicates that the alternative nitrogenases form a monophyletic lineage that is nested among Mo-nitrogenase, indicating that alternative nitrogenases are derived from Mo-nitrogenase (Boyd et al., 2011b). While the phylogenetic studies described in the chapter are limited by only being able to analyze sequenced extant organisms, they provide a compelling case for Mo-nitrogenase emerging prior to alternative nitrogenase. The branching order of alternative nitrogenase (i.e., the nesting of this lineage among strictly anaerobic taxa) further suggests that the differentiation in metal usage in the nitrogenase isoforms is likely to have occurred in an anoxic environment.

### What is the nature of the metal complement of “uncharacterized nitrogenases”?

The aforementioned phylogenetic studies delineate the metal composition of nitrogenase homologs by phylogenetic clustering with proteins from organisms for which their nitrogenase has been characterized to varying extents. Recently, a number of nitrogenase homologs that form a deep branching monophyletic lineage [albeit still derived from Mo-nitrogenase (Boyd et al., 2011b)] have been identified (Figure 2). These nitrogenase homologs form a novel lineage that does not harbor representative sequences for which biochemical information about the active site cluster exists (Boyd et al., 2011b; Dos Santos et al., 2012), precluding assignment of the metallic composition of their active site clusters (hence, “uncharacterized nitrogenase”). Some of these uncharacterized nitrogenase gene clusters don't obey our established criteria (i.e., requirement to encode for homologs of *nifHDKENB*) and instead comprise only *nifHDKEB* (Boyd et al., 2011b). Nonetheless, isotopic tracer experiments suggest that organisms harboring uncharacterized nitrogenase are capable of incorporating N_2_ into biomass (Mehta and Baross, 2006; Dekas et al., 2009).

Key differences in the active site cofactor protein environment of the different metal containing nitrogenases can be used to classify the hypothetical metal composition of nitrogenase homologs that have not been characterized biochemically. As mentioned previously, alternative nitrogenases are less efficient as N_2_ reduction catalysts and previous biochemical studies have also shown interesting differences in the other catalytic properties (Eady, 1996). Alternative nitrogenases produce a larger proportion of hydrogen as a product in the nitrogenase reaction when compared to Mo-nitrogenase. Acetylene reduction catalyzed by the Mo-nitrogenase results in ethylene as the sole product in contrast to the alternative nitrogenases that produce detectable quantities of ethane in addition to ethylene. The recent observation that nitrogenases are capable of hydrocarbon production with carbon monoxide as a substrate (Lee et al., 2010; Hu and Ribbe, 2008) indicates that Mo-nitrogenases and V-nitrogenases have differing catalytic efficiencies for hydrocarbon production with V-nitrogenase having higher catalytic rates. Interestingly, with respect to these observations, it has been shown that simple site-specific amino acid substitutions of Mo-dependent nitrogenase can affect increased hydrocarbon production from carbon dioxide on the order of that observed for the carbon monoxide dependent hydrocarbon production catalyzed by the V-dependent nitrogenase (Yang et al., 2012). These results indicate that the combination of the metal content and cofactor protein environment that make up the structural determinants account for the subtle differences in substrate reduction properties of the different metal-dependent nitrogenases.

Recently, we conducted a fairly exhaustive study of the polypepetide environment of the deeply rooted uncharacterized nitrogenase based on homology models (Mcglynn et al., 2012). This work clearly indicated that the uncharacterized nitrogenases are more likely to be Mo-dependent nitrogenases than V- or Fe-dependent nitrogenase. Given the organisms that possess uncharacterized nitrogenases occupy anaerobic niches, these findings are in line with our previous studies indicating that the oldest extant nitrogen-fixing organisms are anaerobes and that biological nitrogen fixation had its origins in an anoxic environment. In addition, the observation that these more deeply rooted uncharacterized nitrogenases are likely to be Mo-dependent further supports our previous observations indicating that Mo-dependent nitrogenase are ancestral to the alternative nitrogenases.

### Is there evolutionary relevance to nitrogenase promiscuity?

The ability of nitrogenase to reduce other substrates such as acetylene, cyanide and their ability to convert carbon monoxide and carbon dioxide to hydrocarbon products has enticed some to propose that nitrogenase may have its evolutionary roots in one or more of these catalytic activities (Hu et al., 2011). This is an interesting idea considering that the barrier for these reactions are lower which in turn might afford a stepwise path to achieving an enzyme capable of overcoming the enormous activation barrier of N_2_ activation. However, homologs of nitrogenase that have the dedicated function of specifically reducing these types of substrates or analogous compounds *in vivo* have not been identified in extant biology and it is difficult to envision conditions that would have a strong selective pressure for such processes. The recent observation that Mo- and V-dependent nitrogenases are capable of hydrocarbon production from carbon monoxide has been used to suggest a link between nitrogenase dependent production of reduced carbon and nitrogen (Hu et al., 2011). Although an intriguing idea, it is hard to imagine a selective pressure that would select for such a complicated and ATP dependent mechanism for generating reduced carbon early during life's evolution, especially in light of the presence of other viable mechanisms of carbon reduction (e.g., CO or CO_2_) (Ragsdale and Pierce, 2008; Fuchs, 2011). The Wood-Ljungdhal pathway is a prime example of a mechanism that was likely to already exist when nitrogenase evolved (Martin and Russell, 2007; Poehlein et al., 2012; Nitschke et al., 2013) and the key enzyme, the carbon monoxide dehydrogenase/acetyl CoA synthase, for example has a much more common occurrence in deeply rooted microorganisms than nitrogenase and is by all accounts a more ancient enzyme. We propose that *i*) nitrogenase evolved in response to selective pressure of fixed nitrogen availability and *ii*) nitrogenase promiscuity and the ability to reduce other substrates is a product of evolving a redox enzyme that can overcome the largest activation barrier in biology (Rees, 1993).

### What is the evolutionary origin of nitrogenase?

**Figure 4:**
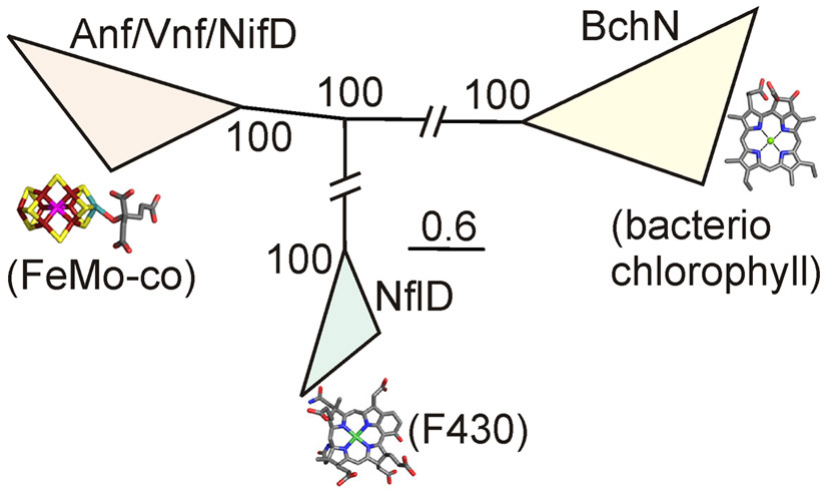
**Phylogenetic relationships between Anf/Vnf/NifD, Bch/ChlN, and NflD proteins, as reproduced from Boyd et al. (2011b).** Parsimony would suggest that the ancestor of this paralogous group of proteins likely harbored an open active site cavity similar to that which is present in modern NflD or Chl/BchN proteins. The ancestor of these protein complexes (at the trifurcation point of the tree) likely encoded a single structural protein approximating NflD. A series of ancient duplications followed by independent evolution yielded the precursor to the heterotetrameric BchNB and NifDK complex (See Figure 3 for a schematic outlining this evolutionary trajectory).

The question of the ancestor of nitrogenase is intriguing and is framed by numerous paradigms that are not strongly supported by empirical observations gleaned from extant biology. In today's world, the availability of fixed nitrogen limits global nutrition and productivity (Falkowski, 1997); it is likely that this anthropogenic-centered focus leads to the tendency to place the emergence of biological nitrogen fixation as a very early event and perhaps even a primordial process. However, even the simplest evolutionary observations such as the aforementioned limited association of biological nitrogen fixation in deeply rooted lineages are not consistent with this process being a property of LUCA. The history of nitrogen availability is not something that can, as of yet, be ascertained from the geologic record so the true time at which selective pressure was sufficient to affect the emergence of such a complicated biochemical process is unclear. Some insights, however, can be assembled from evolutionary relationships of nitrogenase with other paralogous protein complexes associated with chlorophyll and bacteriochlorophyll biosynthesis (Burke et al., 1993; Xiong et al., 2000; Bröcker et al., 2008) and the related enzyme complex proposed to be involved in cofactor F_430_ biosynthesis present in methanogens (Raymond et al., 2004; Staples et al., 2007; Boyd et al., 2011b) (Figures 3, 4).

There is an emerging body of work on the biochemistry of the dark operative protochlorophyllide reductase complex involved in bacteriochlorphyll biosynthesis (Fujita and Bauer, 2000; Bröcker et al., 2008, 2010; Sarma et al., 2008; Muraki et al., 2010). In brief, the enzyme catalyzes the stereo-specific reduction of the C17–C18 double bond of the D-ring of protochlorophyllide to form chlorophyllide. Presumably the nature of this stereo-specific reduction is facilitated by an analogous gated electron transfer mechanism required in biological nitrogen fixation and thus involves an analogous enzyme complex having a homolog of the nitrogenase Fe protein, BchL, and a MoFe protein analogous component, BchNB. Whereas NifDK harbors a cavity where FeMo-co binds, BchNB possesses a cavity where protochlorophyllide binds and where substrate reduction occurs (Bröcker et al., 2010; Muraki et al., 2010), the latter of which involves BchL-dependent electron transfer reactions to affect substrate reduction (Sarma et al., 2008).

Relating the arguably simpler protochlorophyllide reductase to the more complex cofactor-containing nitrogenase from a structural perspective, parsimony would invoke that the simplest structure is the evolutionary ancestor (Figure 3). That is to say the simplest evolutionary trajectory is one in which the common ancestor approximates the structure of the protochlorophyllide reductase (Bröcker et al., 2010; Muraki et al., 2010) or a cofactor-less nitrogenase (Schmid et al., 2002). The mechanism of cofactor biosynthesis is an additional source of insight when thinking of plausible scenarios for the evolution of nitrogenase. The final step in nitrogenase enzyme maturation is in fact the insertion of a preformed cofactor (on NifEN) into a cofactor-less nitrogenase (NifDK) (Rubio and Ludden, 2005, 2008; Hu and Ribbe, 2008) that for all intents and purposes approximates the salient structural features of the protochlorophyllide reductase (BchNB) (Figure 4). Interestingly, neither the cofactor-less nitrogenase nor the cofactor on its own have nitrogen reducing activity, observations that when considered in the context of evolutionary history of the genes required to synthesize an active nitrogenase (see above) narrow down viable scenarios. The most parsimonious scenario involves a common ancestor with an open cavity that resembles the protochlorophyllide reductase serendipitously binding a modified iron-sulfur cluster fragment and the result is a protonitrogenase with a small, albeit highly selectable, level of nitrogen reducing activity (Soboh et al., 2010; Boyd et al., 2011b). The cluster fragment could be in the form of a radical SAM modified carbide iron-sulfur cluster similar to the cofactor intermediate formed at an early state in FeMo-cofactor biosynthesis (e.g., NifB-co) (Wiig et al., 2012). In this scenario, the nitrogenase would be under continual selective pressure to improve catalytic efficiencies, which would provide the selective impetus to adapt the active site cluster into FeMo-co through refinement (emergence of *nifEN*) of the complicated biosynthetic pathway observed in extant biology.

### Conflict of interest statement

The authors declare that the research was conducted in the absence of any commercial or financial relationships that could be construed as a potential conflict of interest.

## Key Concept

Last Universal Common Ancestor (LUCA): LUCA is the last common ancestor of all extant life prior to the divergence of archaea and bacteria and is believed to date to ~3.5 to 3.8 billion years ago (bya). Extant proteins that can be mapped back to LUCA through phylogenetic analyses suggest that the functionalities encoded by these genes were properties of life prior to the divergence of archaea and bacteria. Thus, placing proteins as either present or absent in LUCA represents a key mechanism by which evolutionary biologists can place biochemical reactivates in evolutionary time.
Monophyletic/Paraphyletic: Phylogenetic reconstructions of genes or proteins that reveal lineages comprised of just archaea, bacteria, or eukarya, relative to the branching of proteins derived from taxa representing the other domains, are termed monophyletic. Alternatively, phylogenetic reconstructions of genes or proteins that reveal lineages comprised of a mixture of archaea, bacteria, or eukarya, are termed paraphyletic. Monophyletic relationships observed in phylogenetic reconstructions of archaeal and bacterial proteins is suggestive of an origin prior to the divergence of these two domains at LUCA whereas paraphyletic (mixed branching order) relationships of archaeal and bacterial genes or proteins is suggestive of a role of lateral gene transfer in the evolution of a given protein and may suggest an origin for a given biochemical process after the divergence of archaea and bacteria from LUCA.
Lateral Gene Transfer (LGT): LGT, or horizontal gene transfer, of genetic material is a predominant mechanism by which biochemical and metabolic evolution has occurred throughout geological time. A significant fraction of genes encoded by an organism are acquired through vertical transmission, or through the replication of chromosomal DNA and the direct acquisition of this material from a parental ancestor. LGT, on the other hand, represents a mechanism by which genetic material can be attained from organisms that often span phylogenetic boundaries. LGT can occur via the uptake of naked DNA (transformation), viral infection (transduction), or through direct transfer of DNA between two taxa (conjugation). The aforementioned mechanisms often require close spatial proximity of host and recipient cells, which implies an important role for ecology in dictating the distribution of taxa and subsequent LGT events. The latter is clearly demarcated in the evolutionary of nitrogenase. In particular, the phylogenetic relationships noted between nitrogenase genes associated with thermophilic members of the *Aquificales* and thermophilic members of the *Deferribacteres* implies that the acquisition of the ability to fix N_2_ in the *Aquificales* occurred in a high temperature environment where both ancestral populations were present.
Gene duplication: Gene duplication represents a primary mechanism by which molecular evolution and biochemical diversification can occur. These events are random and initially generate identical copies of genes that encode proteins with identical biochemical reactivities. Given that the biochemical functionalities of duplicated proteins are initially redundant, the duplicated genes are under different selective pressure and can evolve to generate novel functionalities that can lead to selectable and novel biochemical phenotypes. The nitrogenase/protochlorophyllide reductase superfamily has undergone numerous gene duplication events that have ultimately led to protein products that are involved in two of the most important biochemical processes on earth: biological N_2_ fixation and photosynthesis.
Parsimony: The concept of parsimony suggests that one should accept the most simple explanation that fits the given evidence. In the case of molecular phylogenetics, this means that the explanation that requires the fewest assumptions and fewest evolutionary changes is the most likely.
Gene fusions: Gene fusions represent a hybrid of two or more previously separate (individual) genes. Gene fusions occur through a variety of random processes that often result in a selectable phenotype, which can render a more efficient biochemical process both in space (co-localization of gene products) or time (both genes are under the same transcriptional regulation). In the case of the gene fusion between the radical SAM domain protein encoded by *nifB* and the carrier protein encoded by *nifX*, it is believed that this genetic event enabled co-localization facilitating more efficient synthesis and transfer of the FeMo-co precursor NifB-co to NifEN.
